# Supplementary material for: Multiplex PCR Detection of Respiratory Tract Infections in SARS-CoV-2-Negative Patients Admitted to the Emergency Department: an International Multicenter Study during the COVID-19 Pandemic
Source: Microbiol Spectr. 2022 Sep 26;10(5):e02368-22. doi: 10.1128/spectrum.02368-22 (PMC9603986; doi:10.1128/spectrum.02368-22)
Supplement: Supplemental file 1 — Supplemental material. Download spectrum.02368-22-s0001.pdf, PDF file, 1.1 MB [file spectrum.02368-22-s0001.pdf]

Center	Country	Institution
**C1**	Romania	National Institute of Infectious Diseases “Prof Dr. Matei.Bals”
**C2**	Germany	University of Tübingen
**C4**	United Arab Emirates	Dubai Health Authority
**C5**	Azerbaijan	Azerbaijan Medical University Hospital
**C6**	Turkey	Acibadem Hospital Istanbul
**C7**		Gazi University Hospital Ankara
**C8**		Inonu University Hospital Malatya
**C10**	Saudi Arabia	King Faisal Specialist Hospital & Research Center-Riyadh
**C11**		King Fahad Armed Forces Hospital
**C14**	South Africa	NICD (National Institute for Communicable Diseases of the National Health Laboratory Service (NHLS))
**C15**		SMU (Sefako Makgatho Health Sciences University)-NHLS
**C16**		Wits VIDA, University of the Witwatersrand
**C19**	Kenya	Getrudes Hospital
**C21**	Tunisia	Hôpital Charles Nicolle
**C22**		Hôpital Bourguiba, Sfax
**C23**		Institut Pasteur, Tunis
**C29**	Italy	Fondazione Policlinico Universitario A. Gemelli IRCCS, Rome
**C31**	France	CHRU Nancy
**C32**		CHU Rennes Pontchalliou
**C33**		CHU Clermont-Ferrand
**C34 bis**		CHU Saint Etienne
**C37**	Spain	Hospital Gregorio Marañón, Madrid
**C38**	Egypt	Assiut University Hospitals, Assiut
**C39**		Saudi German Hospital

Supplementary Table 1. Sites’ countries and institutions involved in the study

Type of targets	Number (%) of targets identified
**Coronavirus OC43**	32 (5.6)
**Coronavirus NL63**	22 (3.9)
**Coronavirus HKUI**	4 (0.7)
**Coronavirus 229E**	4 (0.7)
**Influenza Virus A H3**	8 (1.4)
**Influenza Virus H1-2009**	0 (0)
**Influenza Virus A**	1 (0.1)
**Parainfluenza Virus 1**	4 (0.7)
**Parainfluenza Virus 2**	0 (0)
**Parainfluenza Virus 3**	32 (5.6)
**Parainfluenza Virus 4**	8 (1.4)
Bordetella pertussis	1 (0.1)
Bordetella parapertussis	3 (0.5)

Supplementary Table 2. Viral subtypes and bacterial species detected in patients' respiratory tract samples using the RP2plus assay (total number of targets identified = 563).

Patients' age groups (years)	Detections by specific RP2*plus* assay's targets										
	HRV/EV	HCOV	ADV	PIV	RSV	HMPV	FLU A	FLU B	BT	MPP	Total detections
**≤2**	56	7	18	13	25	14	2	1	1	0	137
**3-10**	74	8	14	8	8	4	2	2	0	0	120
**11-20**	17	0	5	2	0	0	1	1	0	0	26
**21-30**	40	11	3	3	4	1	1	0	0	0	63
**31-40**	43	12	3	2	1	0	1	1	0	0	63
**41-50**	25	7	3	2	0	0	0	1	0	0	38
**51-60**	18	11	1	1	1	0	1	0	0	0	33
**61-70**	22	3	2	7	1	1	1	1	2	1	41
**71-80**	13	1	4	2	1	0	0	0	1	0	22
**81-90**	7	0	1	2	0	0	0	0	0	0	10
**>90**	1	0	0	3	0	0	0	0	0	0	4
**Missing**	2	2	2	0	0	0	0	0	0	0	6
**Total**	318	62	56	45	41	20	9	7	4	1	563

Supplementary Table 3. Numbers of RP2plus assay detected viruses and bacterial species stratified by patients' age groups. No data were available for 60 patients (all from one hospital site). HRV/EV, human rhinovirus/enterovirus; HCOV, coronavirus; ADV, adenovirus; PIV, parainfluenza virus; RSV, respiratory syncytial virus; HMPV, human metapneumovirus; FLU A, influenza A virus; FLU B, influenza B virus; BT, Bordetella pertussis/B. parapertussis; MPP, Mycoplasma pneumoniae.

Target	Group	Positive	Negative	Chi square, P-value
**HRV/EV**	Adults	169	749	<0.0001
	Children	130	66	
**HCOV**	Adults	45	873	0.1214
	Children	15	181	
**ADV**	Adults	17	901	<0.0001
	children	32	164	
**PIV**	Adults	22	896	<0.0001
	Children	21	175	
**RSV**	Adults	8	910	<0.0001
	Children	33	163	
**HMPV**	Adults	2	916	<0.0001
	Children	18	178	

Supplementary Table 4. Contingency table analysis. Groups of patients with positive or negative RP2 assay's results were compared according to the viral targets listed. Of note, the age group of 11–20 years was removed from the chi-square comparison between children' and adults' groups. HRV/EV, human rhinovirus/enterovirus; HCOV, coronavirus; ADV, adenovirus; PIV, parainfluenza virus; RSV, respiratory syncytial virus; HMPV, human metapneumovirus.

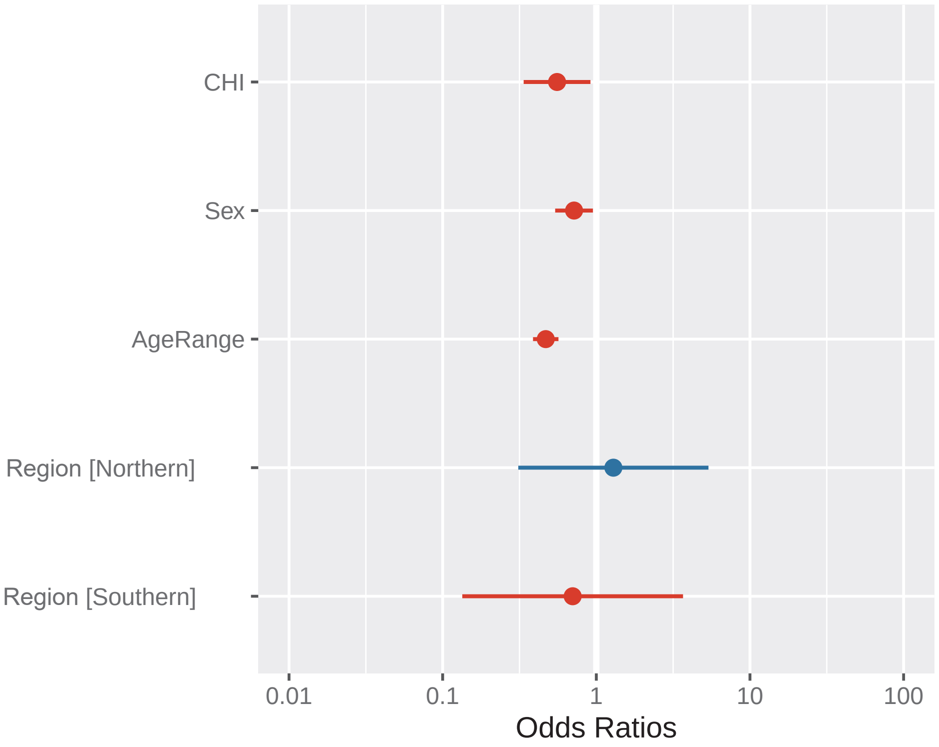

Supplementary figure 1: Effect of country's containment policy (i.e., CHI), sex, age, or seasonality on the probability of a SARS-CoV-2 negative patient to test positive with the RP2 assay.
A logistic regression model, which was built without center C14 data to evaluate potential association between variables, showed an impact of CHI, sex, or age, but not of the regions, on the RP2 assay's positivity rate.

PI	Affiliations
Maya Habous	Head of Microbiology &TB unit, Pathology department, Rashid Hospital, Dubai, United Arab Emirates.
Laila Dabal	Head of Infectious disease unit, Medical Affairs Department, Rashid Hospital, Dubai, United Arab Emirates
Meltem Kilercik	Mehmet Ali Aydinlar Univercity, Medical Faculty, Clinical Biochemistry Department, Istanbul, Turkey
Neval Yurttutan Uyar	Mehmet Ali Aydınlar Univercity, Medical Faculty,Clinical Microbiology Department, İstanbul Turkey
Gulendam Bozdayi	Gazi University, School of Medicine, Department of Medical Microbiology, Head of Division of Medical Virology, Manager of COVID-19 Lab., Ankara/Turkey
Kayhan Caglar	Gazi University, School of Medicine, Head of Department of Medical Microbiology, Division of Medical Virology, Ankara/Turkey
Baris Otlu	Inonu University Medical Faculty. Medical Microbiology Department, Malatya, Turkey
Yusuf Yakupogullari	Inonu University Medical Faculty. Medical Microbiology Department, Malatya, Turkey
Iskender Karalti	Central Laboratory, Therapeutic educational Clinic, Medical University of Azerbaijan, Azerbaijan<br>Faculty of Health Sciences Yeditepe University
Bayram Tagiyev	Central Laboratory, Therapeutic educational Clinic, Medical University of Azerbaijan, Azerbaijan
Reem S. Almaghrabi	King Faisal Specialist Hospital & Research Center (KFSHRC), Riyadh, Saudi Arabia
Sahar Althawadi	King Faisal Specialist Hospital & Research Center (KFSHRC), Riyadh, Saudi Arabia
Mohammad Ghazi Qasem	King fahd armed forces hospital, Jeddah, Saudi Arabia
Abdulwahab Alzahrani	King fahd armed forces hospital, Jeddah, Saudi Arabia
Adrian Streinu-Cercel	National Institute of Infectious Diseases "Prof. Dr. Matei Bals" Dr. Calistrat Grozovici #1, 021105 Bucharest, Romania<br>Infectious Diseases Department - Carol Davila Medicine and Pharmacy University Bucharest, Romania
Anca Streinu-Cercel	National Institute of Infectious Diseases "Prof. Dr. Matei Bals" Dr. Calistrat Grozovici #1, 021105 Bucharest, Romania<br>Infectious Diseases Department - Carol Davila Medicine and Pharmacy University Bucharest, Romania
Evelyne Schvoerer	Laboratoire de Virologie, CHRU de Nancy Brabois, F-54511 Vandoeuvre lès Nancy, France<br>Laboratoire de Chimie Physique et Microbiologie pour les Matériaux et l'Environnement, LCPME UMR 7564 CNRS-UL, F-54600, Villers-lès-Nancy, France
Cédric Hartard	Laboratoire de Virologie, CHRU de Nancy Brabois, F-54511 Vandoeuvre lès Nancy, France<br>Laboratoire de Chimie Physique et Microbiologie pour les Matériaux et l'Environnement, LCPME UMR 7564 CNRS-UL, F-54600, Villers-lès-Nancy, France
Vincent Thibault	Univ Rennes, Department of Virology, CHU Rennes, Inserm, EHESP, Irset (Institut de recherche en santé, environnement et travail) - UMR_S 1085, F-35000 Rennes, France
Charlotte Pronier	Univ Rennes, Department of Virology, CHU Rennes, Inserm, EHESP, Irset (Institut de recherche en santé, environnement et travail) - UMR_S 1085, F-35000 Rennes, France
Cécile Henquell	CHU Clermont-Ferrand, 3IHP, Virology Department, Clermont-Ferrand, France<br>Clermont Auvergne Université, CNRS UMR 6023, LMGE, Clermont-Ferrand, France
Amélie Brebion	CHU Clermont-Ferrand, 3IHP, Virology Department, Clermont-Ferrand, France
Sylvie Pillet	Laboratoire des agents infectieux et d'hygiène, Plateau de Biologie, CHU de Saint-Etienne, Saint-Etienne, France
Rémi Labetoulle	Laboratoire des agents infectieux et d'hygiène, Plateau de Biologie, CHU de Saint-Etienne, Saint-Etienne, France
Peter Silke	Institute of Medical Microbiology and Hygiene, University of Tübingen, Elfriede-Aulhorn-Str. 6, 72076, Tuebingen, Germany
Tina Ganzenmueller	Institute for Medical Virology, University of Tübingen, Elfriede-Aulhorn-Str. 6, 72076, Tuebingen, Germany
Kristina Schmauder	Institute of Medical Microbiology and Hygiene, University of Tübingen, Elfriede-Aulhorn-Str. 6, 72076, Tuebingen, Germany
Patricia Munoz	Clinical Microbiology and Infectious Diseases, Hospital General Universitario Gregorio Marañón, Madrid, Spain<br>Instituto de Investigación Sanitaria Hospital Gregorio Marañón, Madrid, Spain<br>Medicine Department, School of Medicine, Universidad Complutense de Madrid, Madrid, Spain<br>CIBER Enfermedades Respiratorias- CIBERES (CB06/06/0058), Madrid, Spain
Almudena Burillo Albizua	Clinical Microbiology and Infectious Diseases, Hospital General Universitario Gregorio Marañón, Madrid, Spain<br>Instituto de Investigación Sanitaria Hospital Gregorio Marañón, Madrid, Spain<br>Medicine Department, School of Medicine, Universidad Complutense de Madrid, Madrid, Spain
Beatrice Kabera	Department of Clinical Pathology, Gertrudes Children Hospital, Nairobi, Kenya.
Janet Maranga	Department of Clinical Pathology, Gertrudes Children Hospital, Nairobi, Kenya.
Nicole Wolter	Centre for Respiratory Diseases and Meningitis, National Institute for Communicable Diseases (NICD) of the National Health Laboratory Service, Johannesburg, South Africa<br>School of Pathology, Faculty of Health Sciences, University of the Witwatersrand, Johannesburg, South Africa
Mignon du Plessis	Centre for Respiratory Diseases and Meningitis, National Institute for Communicable Diseases (NICD) of the National Health Laboratory Service, Johannesburg, South Africa<br>School of Pathology, Faculty of Health Sciences, University of the Witwatersrand, Johannesburg, South Africa
Temitayo Famoroti	National Health Laboratory Service (NHLS), Sefako Makgatho Health Sciences University (SMU), Department of Clinical Virology, South Africa
Jeannette Wadula	Department of Clinical Microbiology & Infectious Diseases, National Health Laboratory Services, School of Pathology, CH Baragwanath Academic Hospital, University of Witwatersrand, Faculty of Health Sciences
Marta C. Nunes	South African Medical Research Council, Vaccines and Infectious Diseases Analytics Research Unit, Faculty of Health Sciences, University of the Witwatersrand, Johannesburg, South Africa;<br>Department of Science and Technology/National Research Foundation, South African Research Chair Initiative in Vaccine Preventable Diseases, Faculty of Health Sciences, University of the Witwatersrand, Johannesburg, South Africa
Hebatallah Gamal Rashed	Professor of Clinical Pathology, Assiut University, Assiut, 71111, Egypt
Maha Mohamed Elkholy	Professor of Chest disease, Assiut University, Assiut, 71111, Egypt
Mohamed Basiouny Yahia	Lecturer of clinical pathology alazhar university, SGH laboratory director, Egypt
Nevine Abd Elfattah	Professor of Pulmonology Faculty of Medicine, Ain Shams University and Head of pulmonary unit at SGH Cairo, Egypt
Asma Ferjani	Charles Nicolle Hospital, Laboratory of Microbiology, Tunis,Tunisia<br>University of Tunis El Manar, Faculty of Medicine, LR99ES09, Tunis, Tunisia
Ilhem Boutiba-Ben Boubaker	Charles Nicolle Hospital, Laboratory of microbiology, Tunis,Tunisia<br>University of Tunis El Manar, Faculty of Medicine, LR99ES09, Tunis, Tunisia
Adnane Hamammi	Chef de Service Microbiologie, CHU Habib BOURGUIBA, Sfax, Responsable du Laboratoire Bactériologie Virologie, Faculté de Médecine de Sfax
Hèla Karray Hakim	Service de Microbiologie, CHU Habib BOURGUIBA, Sfax, Laboratoire Bactériologie Virologie, Faculté de Médecine de Sfax
Mariem Gdoura	Laboratory of Clinical Virology, WHO Regional Reference Laboratory for Poliomyelitis and Measles for the EMR, Institut Pasteur de Tunis, University of Tunis El Manar, 13 place Pasteur, BP74 1002 le Belvédère, Tunis, Tunisia.<br>LR20IPT10 Laboratory of Virus, Host and Vectors, Institut Pasteur de Tunis, University of Tunis El Manar, Tunis, Tunisia.<br>Faculty of pharmacy of Monastir, University of Monastir
Triki Henda	Laboratory of Clinical Virology, WHO Regional Reference Laboratory for Poliomyelitis and Measles for the EMR, Institut Pasteur de Tunis, University of Tunis El Manar, 13 place Pasteur, BP74 1002 le Belvédère, Tunis, Tunisia.<br>LR20IPT10 Laboratory of Virus, Host and Vectors, Institut Pasteur de Tunis, University of Tunis El Manar, Tunis, Tunisia.
